# Effect of Visual Feedback on the Eye Position Stability of Patients with AMD

**DOI:** 10.3390/vision3040059

**Published:** 2019-11-04

**Authors:** Esther G. González, Mark S. Mandelcorn, Efrem D. Mandelcorn, Luminita Tarita-Nistor

**Affiliations:** 1 Krembil Research Institute, Toronto Western Hospital, Toronto, ON M5T 2S8, Canada; lumi.tarita-nistor@rogers.com; 2 Department of Ophthalmology & Vision Sciences, University of Toronto, Toronto, ON M5T 2S8, Canada; mark.mandelcorn@utoronto.ca (M.S.M.); efrem.mandelcorn@utoronto.ca (E.D.M.); 3 Centre for Vision Research, York University, Toronto, ON M3J 1P3, Canada

**Keywords:** fixation stability, eye position stability, visual feedback, age-related macular degeneration, central vision loss

## Abstract

The sources of the reduced fixation stability exhibited by patients with central vision loss in the light are relatively well understood, but we have no information on how they control eye position in complete darkness, in the absence of visual error signals. We therefore explored the effect of visual feedback on eye position stability by testing patients with age-related macular degeneration (AMD) and controls with normal vision in the light and in complete darkness. Nine patients (ages 67 to 92 years) and 16 controls (ages 16 to 74 years) were tested binocularly in the light and in complete darkness while remembering the location of a now invisible target. Binocular eye position was recorded with a video-based eye tracker. Results show that eye position stability both in the light and in the dark is worse for patients than for controls and that, for the two groups, eye position stability in the dark is, on average, 5.9 times worse than in the light. Large instability of fixation in patients with AMD was found even in absolute darkness when the scotoma cannot impair vision. These data reflect permanent changes in the oculomotor reference of patients with AMD.

## 1. Introduction

Age-related macular degeneration (AMD) is a progressive maculopathy that damages the photoreceptors and can lead to the loss of central vision [1,2]. This loss has detrimental effects on basic visual functions such as acuity, contrast sensitivity, and colour vision [3,4,5] and, consequently, on the higher order visual and oculomotor functions that are based on them [6,7,8,9]. The habitual use of an area of eccentric retina called the preferred retinal locus or PRL allows patients to adapt to the damage [10,11,12], but fixation instability is among the oculomotor consequences stemming from eccentric viewing. Fixation stability in patients with central vision loss has been examined to a large extent [13,14,15,16,17], but the purpose of this study was to explore the effects of visual feedback on its control.

Rather than noise, microsaccades and drifts—the movements of the eyes made while we maintain eye position on a target—are now considered essential for the visual processing of fine detail [18] and for maintaining the binocular coordination that results in stable perception [19]. The third class of eye movement, tremor, has received less attention due to the technical challenges involved in its measurement. Control of eye position in the light is served by the same receptive field organization underlying visual acuity and is accomplished by means of a visual error signal in relation to the centre of fixation. 

The stability of fixation makes use of a feedback loop that continually compares an actual to a target condition and corrects fixation based on the resulting error signal. The control of eye position makes use of two kinds of error signals: (1) the visual error produced by the position of the visual target on the retina, and (2) the non-visual extraretinal signals produced by the control of the position of the eye in orbit [20]. For targets falling near or within the foveal area, eye position stability depends on the visual error produced by the distance between the target’s location on the retina and the centre of fixation. Given the accuracy of this signal in people with healthy foveae [21], extraretinal signals are ignored, but as the visual error becomes progressively less accurate away from the fovea [17,22], extraretinal signals become more important for maintaining the eyes stable. During fixation, these mechanisms have continuous control over drifts rather than over microsaccades because the latter have durations too short in relation to the lag of visual feedback [23].

In the absence of visual feedback, extraretinal signals provide information for controlling the eyes’ position in the dark by comparing the innervation pattern (outflow) to the extraocular muscles used to fixate visible targets [24], and from the proprioceptive signals arising from the stretch receptors in the extraocular muscles (inflow) [25,26,27]. Although both sources of information have been shown to be used in eye position control, inflow may be better conceived as a long-term calibrator from which outflow derives instantaneous measures of eye position [27]. Whereas a target’s position is defined relative to the physical environment, stabilizing eye position without error signals in absolute darkness makes use of a memorized position of the eye relative to the orbit as well as the innervation to the extraocular muscles.

We have no information as to how central vision loss affects the control of eye position in complete darkness, in the absence of visual error signals. The purpose of this study was to compare the eye position stability of patients with central vision damage to that of controls with healthy vision when tested in the light and in complete darkness; that is, when under non-visual extraretinal control. Our hypothesis was that fixation stability in complete darkness should be similar for both groups because the reduced vision of the AMD group does not play a role in controlling eye position. This, however, would only hold if in complete darkness patients returned their eyes to primary position. If instead of returning to primary position in complete darkness patients maintain the position of the eyes in orbit that they exhibit while fixating in the light—an effect we tested—, this would be evidence of a permanent change in their oculomotor reference [9] that would likely result in reduced fixation stability as a result of the eyes being away from primary position.

The data presented here were collected along with those in an already published study of the active horizontal vestibulo-ocular reflex (VOR) of patients with AMD and controls performed at a frequency of 0.5 Hz [28]. The VOR is an image stabilization reflex that minimizes the motion of a target on the retina during head movement by producing compensatory eye movements in the direction opposite to those of the head. That study, like the present one, tested the VOR in the light with a visible target and in complete darkness using a target whose location participants had to remember and could no longer see; that is, under visual and non-visual control.

## 2. Materials and Methods

This research was approved by the Research Ethics Board of the University Health Network and conducted in accordance with the tenets of the Declaration of Helsinki (project code 14-8291). Patients were recruited from the Eye Clinic at the Toronto Western Hospital and controls were students, volunteers, and hospital staff. All participants provided written informed consent.

### 2.1. Participants

*AMD group.* This group consisted of 9 patients (2 females) with a confirmed diagnosis of bilateral AMD ranging from early to late according to the Beckman Initiative Classification [29]. They all had no other neurological or ocular pathologies with the exception of mild cataracts (2+ or less in a four-point grading system modified from LOCS III [30]) and were under treatment for neovascular AMD, or monitored for disease progression for intermediate AMD. Their mean acuity for the better eye was 0.46 (SD = ±0.25) logMAR and for the worse eye 0.98 (SD = ±0.44) log MAR. Their stereoscopic thresholds ranged from 400 arc s^−1^ to nil and their ages ranged from 67 to 92 years (mean = 78.89, SD = ±8.82). By coincidence, the right eye was the eye with the better acuity for all the patients. Table 1 includes the clinical and demographic characteristics of the two groups.

### 2.2. Equipment

Horizontal and vertical eye positions were recorded binocularly with a head-mounted video-based infrared eye tracking system (Series 2020; El-Mar, Inc., Toronto, ON, Canada). with a sampling rate of 120 Hz, a maximum resolution of 6 min arc, and linearity over a range of at least 30 deg in the horizontal and 25 deg in the vertical meridians. The accuracy and precision of this system have been found to be comparable to those of a magnetic search coil system [31]. The El-Mar system records head position with a single receiver mounted on a frame and placed near the centre of rotation of the head. The receiver uses a pulsed magnetic field transmitted from an earth-fixed unit at a rate of 60 Hz and records head position (x, y, z) and orientation (azimuth, elevation, roll) in 3 dimensions within a 1 m^3^ space, approximately [32]. In the sagittal plane, the distance from the centre of rotation of the receiver to the centre of the eyes’ rotation is about 10 cm, depending on the participant’s head.

A graphics and psychophysical testing software, VPixx (VPixx Technologies, Inc., Montreal, QC, Canada), was used to generate the 14 calibration targets, the fixation target, and the 1 s auditory prompts that indicated the end of the eye position trials and the upcoming VOR trials 3 s later. The calibration targets were 9-cycle black and white square-wave radial gratings 3 deg in diameter which have been shown to produce fixation stability that is independent of the visual acuity of patients with AMD [33]. A 1 deg white dot with a brightness of 122 cd/m^2^ was the fixation target shown on a black background of 2.7 cd/m^2^.

The stimuli were seen at a viewing distance of 57 cm on a Samsung monitor (Sync Master 900 NF; Samsung, Seoul, South Korea) with a 34.4 × 26 cm field of view, a refresh rate of 120 Hz, and a 1024 × 768 pixel resolution. The interface between the Macintosh display computer and the recording eye tracker was a Chromatrig graphics card (VPixx Technologies, Inc., Montreal, QC, Canada). During calibration only, the participants were seated with their head steadied by a chinrest in a custom-made chair that brought the eyes to primary position when looking at the center of the monitor. For the patients, in addition to low fixation variability, a successful calibration meant that they had used the same PRL for fixating the centre of all the targets. After calibration, the chinrest was removed in anticipation of two VOR trials, one in the light and one in complete darkness. No optical correction was worn during eye movement recording but the size and contrast of the calibration and fixation stimuli were above threshold for both groups.

### 2.3. Procedure

Each VOR trial began with a fixation stability task that lasted 15 s, after which the computer provided a 1 s warning tone followed, 3 s later, by the VOR task lasting 20 s. The end of the VOR was marked by a 1 s warning tone and, 3 s later, by a second 15 s fixation stability task. In this paper we present only the fixation stability data from the beginning of the two VOR trials (i.e., the first 15 s).

Participants were instructed to keep the head as still as possible during the fixation stability tasks. Before the trial in complete darkness, participants were shown the visual target before it was covered and were instructed to maintain the head steady and the eyes on the now invisible target’s memorized location. The various elements of the experimental trials were repeated as necessary in order to ensure everyone understood the tasks and the meaning of the auditory prompts. Viewing was binocular.

Because corrective spectacles affect the gain of the VOR [34,35], participants removed their optical correction at least 1 h before testing. After the eye movement recording, visual acuity was obtained with a computerized, high contrast version of the Early Treatment of Diabetic Retinopathy Study (ETDRS) test at 6 m with participants wearing their spectacles for distance viewing. Stereoscopic acuity was then measured using the Fly Stereotest (available in the public domain at http://www.stereooptical.com) (Table 1).

The trial in the light was run under standard fluorescent lighting and instructions and test trials were always run with the lights on. For the trial run in the dark, our windowless laboratory was in complete darkness. Gaps between the doors and their frames were taped with black foam and all electric equipment was covered with thick black felt. The short duration of the trial in the dark (58 s) ensured that the participants were not dark-adapted.

### 2.4. Data Analysis

The measure of eye and head position stability was a bivariate contour ellipse area (BCEA) with a probability value of 68.2%. Custom software written in Matlab™ (Matlab; The MathWorks, Natick, MA) was used to remove, offline, any blinks, glitches or recording errors and to compute the BCEA according to the following:

$$\mathrm{BCEA}\;=\;\pi \;\chi^{2}\;{\mathrm{SD}}_{x}\;{\mathrm{SD}}_{y}\sqrt{1-r^{2}}\;{\;}_{,}$$

where SD_x_ and SD_y_ are the standard deviations of the horizontal and vertical positions, respectively, r is their Pearson product-moment correlation, and χ^2^ = 2.291 is the chi-squared value (2 df) corresponding to a probability value of *p* = 0.682 (±1 SD). In order to normalize the data, statistical analyses were performed on the logarithmically (base 10) transformed BCEA values. Alpha level was set at 0.05 for all the statistical tests.

## 3. Results

### 3.1. Age

All analyses were repeated using age as a covariate which was found to be non-significant and did not alter the conclusions.

### 3.2. Eye Position Stability

For the AMD group, correlations between visual acuity and fixation stability were non-significant (smallest *p* = 0.12). The correlations between fixation stability in the dark and fixation stability in the light were also non-significant, but removing the data from patient P5 who had the largest difference score (logBCEA_dark_ − logBCEA_light_), produced a significant correlation for the better eye, r(6) = 0.71, *p* = 0.02 only. No other significant correlations were found between the clinical or demographical variables and fixation stability.

For the two groups, the conjugate data from the left and the right eyes were averaged [36] and analyzed with a 2 (Viewing Condition: light and dark) x 2 (Group: AMD and control) mixed factorial ANOVA on the log_10_ BCEA values. The results yielded significant effects of Viewing Condition (light and dark), F(1,23) = 65.91, *p* < 0.001, partial η^2^ = 0.74, and Group (AMD and control), F(1,23) = 14.86, *p* < 0.001, partial η^2^ = 0.39. The interaction of Viewing Condition and Group was non-significant.

The difference scores (logBCEA_dark_ − logBCEA_light_) of the fixation stability values of the two groups were compared using an independent groups t-test that was found to be statistically non-significant, t(23) = 1.38, *p* = 0.91. As can be seen in Table 2, there was a remarkable similarity in the difference between fixation stability in the light and in the dark (logBCEA_dark_ − logBCEA_light_): 0.78 (SD = ±0.25) deg^2^ and 0.76 (SD = ±0.56 deg^2^) for the controls and patients, respectively. For the two groups these values meant that, on average, the BCEA in the dark was 5.9 times larger than the BCEA in the light (5.97 times for the control and 5.72 times for the AMD group).

### 3.3. Vertical and Horizontal Eye Position

Difference scores for horizontal (x_light_ − x_dark_) and vertical (y_light_ − y_dark_) eye position exhibited during the fixation stability trials in the light and in the dark were analyzed with a mixed factorial ANOVA which showed no statistically significant changes in horizontal (x) or vertical (y) eye position as a function of Viewing Condition (light and dark) or Group (AMD and control).

### 3.4. Head Stability

In order to verify that movements of the head had not contributed to the patients’ eye position instability relative to the controls’, the head position stability of the two groups was analyzed using the log_10_ BCEA of their head’s projected position on a plane parallel to Listing’s plane. A 2 (Viewing Condition: light and dark) × 2 (Group: AMD and control) mixed factorial ANOVA found no statistically significant effects of the two variables or their interaction. Overall, the mean log_10_ BCEA head position stability was 0.55 deg^2^ (SD = ±0.28) for the patients and 0.47 deg^2^ (SD = ±0.46) for the controls.

## 4. Discussion

This study examined the effects of the visual error signal on the eye position stability of patients with AMD by means of two 15 s fixation tasks performed, one in the light and the other in complete darkness. The results showed that the fixation stability of the AMD group was worse than that of the controls both in the light and in the dark and that fixation stability is impaired (i.e., produces larger BCEAs) in the dark, in both groups, when compared with the light condition. The data can be summarized as showing: (1) that changes in the oculomotor reference of patients with AMD can be seen even in complete darkness and, (2) that the difference between visual and non-visual error signals for maintaining eye position stability is a ratio that is similar for patients and controls with normal vision.

Poorer eye position stability of the patients is evident even while under the exclusive control of non-visual extraretinal signals; that is, in complete darkness when the damage to central vision has no visual consequences. We also found that the eyes of the patients do not return to primary position in the dark which means that the central nervous system maintains the eye rotation that allows for eccentric viewing (i.e., the PRL) even when scotomata cannot impair vision. This effect was also found in the VOR data collected from the same patients [28] and is congruent with data reported by White and Bedell [9] in a study of the oculomotor reference of people with bilateral macular disease who tend to report looking “straight ahead” when using the PRL—in other words, during eccentric viewing—reflecting permanent changes in the oculomotor reference. Consistent with this, we found a small indication that eye position control in the dark is related to the patient’s eye position control in the light. 

For patients with central vision damage, the visual error signal becomes less accurate as a function of the distance of the PRL from the fovea [16,37]. The present data show that the extraretinal signal information is also worse for eccentric—as opposed to primary—eye position and may be the reason behind the poorer fixation stability in the dark of the patients with AMD. Unbalanced fatigue of the ocular muscles produces an illusion of motion known as the autokinetic effect [38]. Following the eyes’ deviation to an extreme position, fixation of a small lit target in an otherwise darkened room and with the eyes in central position produces an illusory motion of the target. How much is the eccentric eye in orbit position responsible for the poorer position stability in the dark is a topic for future research in both people with normal vision and patients with central vision loss.

Another source of error for the patients with AMD is the loss of stereoscopic vision. In general, disease progression increases the stereoscopic thresholds of the patients leaving, at best, the coarse stereo exhibited by some of the patients studied here and completely destroying it in the others. However, a linear relationship between stereoscopic depth perception and fixation stability is not a perfect one and holds only for cases with similar damage to both eyes, breaking down in cases of patients with large interocular differences. For instance, using a binocular eyetracker and a new method of determining the absolute location of the PRL, we reported a patient who was effectively monocular during binocular viewing because the PRL of the worse eye landed within the scotoma [39]. This is because binocular fixation, like binocular acuity, is driven by the better-seeing eye [36] and the majority of patients with AMD exhibit coordinated movements of the eyes [40].

One major difficulty for studies of binocular vision in patients with central vision loss is that the software of commercially available eyetrackers assumes that the calibration targets are fixated foveally. For patients with damaged central vision, the absolute location of the binocular PRL can be approximated by using the values obtained from scanning laser ophthalmoscopes or microperimeters—both monocular instruments—and adding the resulting distance of the PRL to the estimated location of the former fovea to the calibrated values. The problem with this approach is that the location of the PRL measured monocularly may not be always the same as that used during binocular viewing, as is sometimes found in cases with large interocular acuity/pathology differences [41,42,43].

Our data show that, for controls while under the exclusive control of extraretinal signals, fixation stability is 5.97 times worse than when under the combined use of visual and non-visual feedback. A surprising finding was that for the patients, the visual error signal, in spite of being less accurate than in controls, improves fixation stability by a similar factor of 5.72 when compared to the exclusive control of the non-visual extraretinal signals. Whether this finding is serendipitous or the result of eye movement control mechanisms requires further investigation with a larger patient sample.

In lieu of age-matched controls, the control group in this study included participants with a wide age range. Age, however, was not found to be a significant covariate in our results and the fact that the deficits in eye position control in the patients were a function of pathology rather than age is consistent with previous reports [44,45]. Another effect of the age difference between the groups was the fact that the patients, being over 67 years old, were likely presbyopic while many of the controls were not. Blurred visual targets produced by poor accommodation, however, do not have a significant effect on fixation stability [45,46] and are not likely to have affected our findings. Finally, the sample size in this exploratory study was insufficient for determining the effects of interocular differences in terms of the scotoma’s size, its position on the retina, its shape, density—and we suspect the type of maculopathy—on fixation stability in the dark.

This research is important because understanding how oculomotor control is achieved after the loss of central vision gives us further insight into the normal control of the movements of the eyes and because knowledge of the mechanisms of eye movement control in people with central vision loss is essential for elucidating their adaptation mechanisms and the design of successful rehabilitation strategies. We do not know how brain plasticity affects the non-visual extraretinal control of eye position given that the research done on fixation stability has only made use of visual error signals [47]. Nevertheless, based on the mild relationship between eye position stability in the light and in the dark shown by 8 of the 9 patients, we predict at least some degree of transfer from the plasticity resulting from experience or visual training to the extraretinal control of eye position in the dark. It would be interesting to determine whether the ratio of eye position stability in the light to eye position stability in the dark changes as a function of this plasticity. Finally, it is possible that because of their eccentric fixation, the autokinetic effect of patients with central vision loss may be stronger than in people with normal vision, another factor that should be verified by future research. Regardless of the results, patients should be made aware of the possibility of illusory motion of visible objects in semi-darkness which could lead to disorientation.

## 5. Conclusions

The instability of fixation exhibited by patients with AMD was found even in absolute darkness when the scotoma cannot impair vision. Patients were also found to maintain the eye rotation corresponding to the PRL in the absence of visual signals, which reflects permanent changes in the oculomotor reference as a result of AMD. The absence of visual error correction makes eye position stability in the dark worse than fixation stability in the light, by a factor that appears to be independent of whether the viewer has a healthy fovea or not. Further research is necessary to discern the mechanism behind this ratio.

## Figures and Tables

**Table 1:** Clinical and demographic characteristics of the two groups.

		Acuity (logMAR)		Stereo	Fixation Stability log_10_ BCEA (deg^2^)	
Group	Age	RE	LE	(arc sec^−1^)	Light	Dark
AMD						
	67	0.34	1.00	400	0.43	0.97
	83	0.98	1.00	800	0.95	1.18
	81	0.30	0.44	400	0.01	2.10
	67	0.44	0.86	nil	0.18	1.09
	81	0.70	2.00	3600	0.94	1.42
	77	0.24	0.70	3600	1.27	1.56
	76	0.57	1.20	3600	0.39	0.95
	82	0.40	1.00	nil	0.87	1.58
	96	0.22	0.64	400	0.09	1.07
mean	78.9	0.46	0.98		0.57	1.32
(SD)	(8.8)	(0.25)	(0.44)		(0.45)	(0.38)
Control						
	58	−0.10	0.00	<40	−0.06	0.93
	46	−0.24	−0.24	<40	−0.20	1.04
	23	−0.26	−0.28	<40	0.31	1.19
	28	−0.10	−0.10	<40	<0.00	0.79
	23	0.00	−0.10	<40	0.27	0.99
	73	−0.04	−0.06	<40	−0.11	0.33
	31	−0.22	−0.22	<40	−0.10	0.57
	25	−0.10	−0.30	<40	−0.20	0.77
	16	0.06	0.04	<40	0.09	1.09
	24	−0.22	−0.24	<40	0.29	0.78
	19	−0.10	−0.10	<40	0.22	0.52
	31	−0.26	−0.22	<40	−0.14	0.48
	48	−0.10	−0.06	<40	−0.30	0.61
	23	−0.06	0.10	<40	−0.20	0.85
	74	0.08	0.04	<40	0.38	1.02
	63	−0.10	−0.10	<40	−0.02	0.68
mean	35.9	−0.08	−0.09		0.01	0.79
(SD)	(20.8)	(0.12)	(0.14)		(0.22)	(0.24)

RE = right eye; LE = left eye. Control group. All 16 controls (9 females) had normal or corrected-to-normal visual acuity (mean = −0.11, SD = ±0.12; range −0.28 to 0.08 log MAR) and stereopsis (score ≤ 40 arc s^−1^). Their ages ranged from 16 to 74 years (mean = 37.81, SD = ±19.65).

**Table 2:** Mean eye position stability [log_10_ BCEA (deg^2^)] for the two groups. Values in parentheses are ±1 SDs.

	Group	
Viewing Condition	AMD	Control
Light	0.57 (0.45)	0.01 (0.22)
Dark	1.32 (0.38)	0.79 (0.24)
Mean differences	0.76 (0.56)	0.78 (0.25)
